# Pharmacist-integrated collaborative outpatient care for risk factor control across the cardiovascular-kidney-metabolic spectrum: a systematic review and meta-analysis of randomized trials

**DOI:** 10.3389/fmed.2026.1850534

**Published:** 2026-08-20

**Authors:** Shan Zhao, Wei Xiong, Fuding Liu, Yangyang Xia, Lifang Zhang, Ying Liu

**Affiliations:** 1 Weight Management Clinic, Huangshi Central Hospital, Affiliated Hospital of Hubei Polytechnic University, Huangshi, Hubei, China; 2 Department of Geriatrics, Huangshi Central Hospital, Affiliated Hospital of Hubei Polytechnic University, Huangshi, Hubei, China; 3 Department of Public Health, Huangshi Central Hospital, Affiliated Hospital of Hubei Polytechnic University, Huangshi, Hubei, China; 4 Chronic Disease Management Center, Huangshi Central Hospital, Affiliated Hospital of Hubei Polytechnic University, Huangshi, Hubei, China; 5 Department of Nursing, Huangshi Central Hospital, Affiliated Hospital of Hubei Polytechnic University, Huangshi, Hubei, China; 6 Department of Gastroenterology, Huangshi Central Hospital, Affiliated Hospital of Hubei Polytechnic University, Huangshi, Hubei, China

**Keywords:** blood pressure control, cardiovascular-kidney-metabolic syndrome, glycated hemoglobin, outpatient care, pharmacist-integrated collaborative care, systematic review and meta-analysis

## Abstract

**Background:**

Cardiovascular-kidney-metabolic (CKM) syndrome requires integrated outpatient strategies that address interrelated hemodynamic and metabolic risk. Pharmacist-integrated collaborative care has emerged as a potential model to improve cardiometabolic control across CKM-relevant populations, but the magnitude and consistency of benefit remain uncertain.

**Methods:**

We conducted a systematic review and meta-analysis of randomized trials evaluating pharmacist-integrated collaborative outpatient care versus usual care or control care in CKM-relevant populations. Twenty-one randomized trials were included, spanning hypertension, type 2 diabetes, and mixed CKM populations, predominantly in primary care settings. Random-effects meta-analyses were performed using restricted maximum likelihood estimation with Hartung-Knapp adjustment.

**Results:**

The most robust finding was for clinic systolic blood pressure. Pooling adjusted estimates from 3 trials showed that pharmacist-integrated collaborative care significantly reduced clinic systolic blood pressure compared with usual care (MD − 7.11 mm Hg, 95% CI -9.65 to −4.58; I^2^ = 0.0%). Continuous HbA1c also favored pharmacist-integrated care in 3 trials (MD -1.26, 95% CI -2.36 to −0.16), although heterogeneity was substantial (I^2^ = 89.2%). This glycemic effect was less stable in sensitivity analysis excluding the high-risk Javaid 2019 trial, after which the pooled estimate attenuated to −0.81% (95% CI -2.11 to 0.49). Exploratory target-attainment analyses were directionally favorable but imprecise and were not considered definitive.

**Conclusion:**

Pharmacist-integrated collaborative outpatient care was associated with a clinically meaningful reduction in clinic systolic blood pressure and may improve HbA1c across CKM-relevant populations. The blood-pressure finding was consistent and robust, whereas the glycemic evidence was more heterogeneous and sensitive to study quality. These findings suggest that pharmacist-integrated collaborative care is a promising outpatient CKM risk-reduction strategy, particularly for hemodynamic control, while underscoring the need for more standardized, high-quality trials for metabolic outcomes.

**Systematic review registration:**

https://www.crd.york.ac.uk/prospero/display_record.php?ID=CRD420261363411, identifier CRD420261363411.

## Graphical abstract

**Figure:**
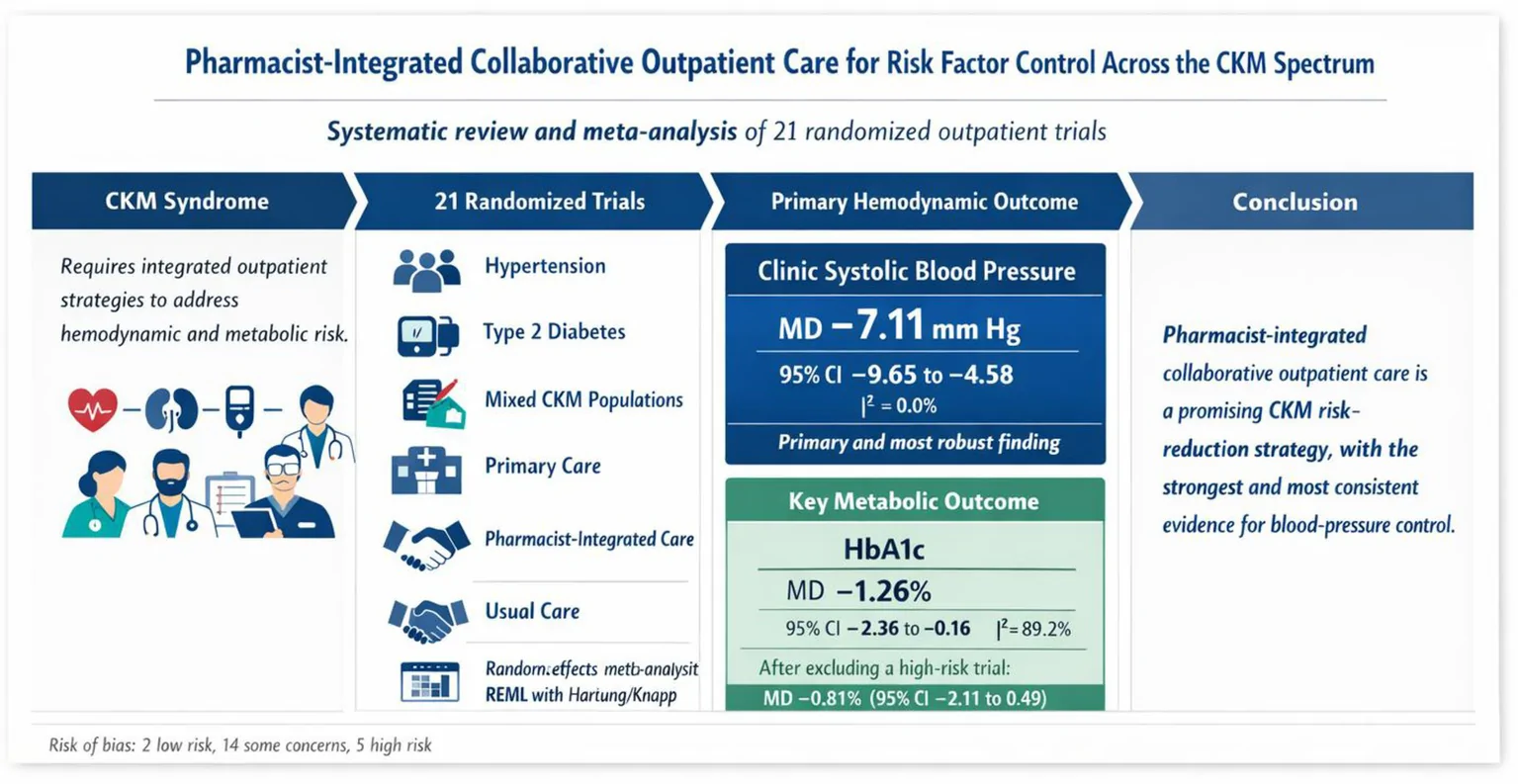

## Introduction

Cardiovascular-kidney-metabolic (CKM) syndrome conceptualizes obesity, insulin resistance, type 2 diabetes, chronic kidney disease, and cardiovascular disease as an interconnected biological continuum rather than a simple aggregation of isolated comorbidities (1). Within this framework, excess adiposity and metabolic dysfunction drive inflammation, oxidative stress, endothelial injury, neurohormonal activation, and progressive target-organ damage, ultimately contributing to both subclinical and overt cardiovascular disease (1). This perspective is closely aligned with contemporary cardiovascular prevention strategies that emphasize earlier risk identification and integrated management of modifiable risk factors across the life course (2). Because dysglycemia and type 2 diabetes are central components of the CKM continuum, accurate identification and classification of diabetes also remain relevant to CKM-oriented prevention and outpatient care (3). The CKM framework therefore has important implications for ambulatory practice, where integrated risk-factor modification must begin long before advanced cardiovascular or renal complications become clinically manifest (1, 2).

Despite contemporary recommendations for blood pressure control and glycemic management, achievement of treatment targets in routine outpatient practice remains suboptimal (4, 5). Persistent gaps in risk-factor control reflect not only disease complexity, but also therapeutic inertia, delayed treatment intensification, medication nonadherence, and fragmented longitudinal follow-up (6–9). These challenges are particularly relevant within CKM syndrome, where multiple hemodynamic and metabolic abnormalities frequently coexist and require coordinated rather than single-disease management approaches (1, 2, 4, 5). Accordingly, there is increasing interest in outpatient care models that can support proactive medication optimization, sustained monitoring, and integrated chronic disease management across CKM-relevant populations.

Pharmacist-integrated collaborative care represents one such strategy. In outpatient and primary care settings, pharmacists may contribute to medication review, therapeutic intensification, adherence support, patient education, home monitoring, and structured follow-up, either through recommendation-based collaboration with physicians or through more protocol-driven care models (10–15). Prior systematic reviews and meta-analyses suggest that pharmacist-led or pharmacist-integrated interventions can improve blood pressure control and other cardiovascular risk factors in ambulatory care (10, 11). More recent studies also support beneficial effects on medication adherence, self-management, safer prescribing, and diabetes-related outcomes across chronic cardiovascular and metabolic populations (12–15). From a CKM perspective, this model is especially attractive because it is scalable within routine outpatient care and capable of targeting several modifiable risk domains simultaneously.

However, the existing evidence base remains fragmented. Previous evidence syntheses have often focused on single conditions such as hypertension or diabetes, evaluated heterogeneous intervention models, or predated the formal CKM framework (10, 11). It remains unclear whether the benefits of these interventions are more consistent for hemodynamic outcomes, such as clinic systolic blood pressure, than for metabolic outcomes, such as glycated hemoglobin, and how robust such findings are when evidence is restricted to randomized outpatient trials. A CKM-oriented synthesis focused specifically on randomized outpatient evidence is therefore needed to clarify the magnitude, consistency, and clinical interpretability of benefit.

We therefore conducted a systematic review and meta-analysis of randomized trials evaluating pharmacist-integrated collaborative outpatient care across CKM-relevant populations, including hypertension, type 2 diabetes, and mixed cardiometabolic populations. Our objective was to quantify the effects of these interventions on major outpatient risk-factor outcomes while distinguishing the most robust findings from exploratory or methodologically limited evidence. By situating pharmacist-integrated care within the CKM framework, this review aims to clarify its potential role as a scalable outpatient strategy for integrated risk reduction across the cardiovascular-kidney-metabolic spectrum.

## Methods

### Study design and reporting

We conducted a systematic review and meta-analysis of randomized trials evaluating pharmacist-integrated collaborative outpatient care across cardiovascular-kidney-metabolic (CKM)-relevant populations. The review was designed to assess the effects of pharmacist-integrated care on major outpatient risk-factor outcomes while distinguishing the most robust quantitative findings from exploratory or methodologically limited evidence. The review protocol was registered in the International Prospective Register of Systematic Reviews (PROSPERO; registration number CRD420261363411). The manuscript was prepared in accordance with the Preferred Reporting Items for Systematic Reviews and Meta-Analyses (PRISMA) 2020 statement (16).

### Eligibility criteria

Studies were eligible if they met the following criteria: (1) randomized trial design, including individually randomized and cluster-randomized trials; (2) adult outpatient populations with hypertension, type 2 diabetes, or mixed cardiometabolic/CKM-relevant risk profiles; (3) pharmacist-integrated collaborative care delivered in primary care, specialty outpatient, or community ambulatory settings; and (4) comparison with usual care, control care, or another nonintegrated care model. Eligible pharmacist interventions included recommendation-based collaborative medication management, protocol-based co-management, structured medication review, adherence support, patient education, and longitudinal follow-up integrated into outpatient care.

We excluded nonrandomized studies, inpatient-only studies, pediatric populations, interventions limited to dispensing or one-off counseling without a collaborative disease-management component, and studies without extractable clinical outcome data relevant to blood pressure, glycemic control, or related cardiometabolic endpoints. Studies with non-usual-care comparators or restricted comparability were retained in the review but reserved for sensitivity, secondary, or narrative analyses where appropriate.

### Information sources and search strategy

We searched PubMed, MEDLINE, Embase, Scopus, and CINAHL from database inception to 18 March 2026. The search strategy combined controlled vocabulary and free-text terms related to pharmacists, collaborative or team-based outpatient care, hypertension, diabetes, and cardiometabolic risk. Database-specific strategies were adapted to the syntax and indexing structure of each source. Only published studies in English were eligible, and there were no search date restrictions. In addition, potentially relevant studies were identified through reference list checking, trial or study register searching, and contact with authors or experts where appropriate. This wording matches the registered PROSPERO record, which lists CINAHL, Embase, MEDLINE, PubMed, and Scopus, specifies English-language published studies only, states no search date restrictions, and includes reference checking, trial/study register searching, and contacting authors or experts as supplementary identification methods.

### Study selection

Records identified through the database searches were screened in two stages. Titles and abstracts were first assessed against the predefined eligibility criteria, followed by full-text review of potentially relevant reports. Multiple reports from the same trial were collated and treated as a single study to avoid double counting. When companion publications or secondary analyses were available, outcome data were extracted from the most informative or methodologically appropriate report for each endpoint.

### Data extraction and management

Data was extracted using a standardized study-level form. Extracted items included country, study design, clinical setting, CKM domain, intervention characteristics, collaborative intensity, medication-management authority, follow-up duration, randomized and analyzed sample sizes, and outcome reporting structure. For quantitative analyses, we extracted outcome-specific data required for pooling, including adjusted study-level effect estimates when reported, corresponding measures of uncertainty, and event counts or continuous summary statistics as appropriate.

When participant counts were inconsistent across the Methods, Results, and participant-flow diagrams, outcome data were preferentially extracted from the analyzed population used in the statistical analyses and outcome tables rather than from the CONSORT flow counts alone. Such discrepancies were documented and considered during risk-of-bias assessment. Studies lacking essential variance data, outcome-level denominators, or sufficiently comparable outcome definitions were retained in the systematic review but excluded from the relevant quantitative pool.

### Outcomes

The principal quantitative outcome for the review was clinic systolic blood pressure, selected as the primary hemodynamic endpoint because it was the most clinically relevant and methodologically consistent outcome across the included outpatient trials. The key metabolic outcome was continuous glycated hemoglobin (HbA1c). Secondary or exploratory outcomes included blood-pressure target attainment, HbA1c target attainment, and additional supportive cardiometabolic outcomes when reported. Outcomes were synthesized quantitatively only when definitions, effect measures, and reporting structures were sufficiently comparable across studies.

### Risk-of-bias assessment

Risk of bias was assessed using the Cochrane Risk of Bias 2 (RoB 2) tool (17). The following domains were considered: bias arising from the randomization process, bias due to deviations from intended interventions, bias due to missing outcome data, bias in measurement of the outcome, and bias in selection of the reported result. Each study received an overall judgment of low risk of bias, some concerns, or high risk of bias. Domain-level judgments were summarized in Supplementary materials, whereas the main manuscript reports the overall distribution of study-level judgments.

### Data synthesis and statistical analysis

Random-effects meta-analyses were performed throughout using restricted maximum likelihood (REML) estimation with Hartung-Knapp adjustment (18). Continuous outcomes were summarized as mean differences (MDs), and binary outcomes were summarized as risk ratios (RRs), each with 95% confidence intervals. Statistical heterogeneity was quantified using the I^2^ statistic.

For the primary clinic systolic blood pressure analysis, adjusted study-level mean differences were preferred whenever available, particularly for cluster-randomized trials or studies reporting covariate-adjusted estimates. These adjusted estimates were pooled using generic inverse-variance random-effects models. For continuous outcomes, final-value analyses and change-from-baseline analyses were not combined in the same pooled model; instead, they were analyzed separately to preserve comparability of effect metrics. Where necessary, the direction of effect was harmonized so that negative values consistently favored pharmacist-integrated collaborative care.

Studies were excluded from a given meta-analysis if they lacked the variance structure required for pooling, did not provide extractable outcome-level denominators, used noncomparable outcome definitions, or involved comparator structures not suitable for the primary usual-care analysis. Such studies remained eligible for narrative synthesis and, where appropriate, for restricted sensitivity or exploratory analyses.

### Sensitivity and exploratory analyses

Prespecified robustness analyses were undertaken to assess the stability of the primary findings. These included sensitivity analyses excluding studies judged at high risk of bias, a leave-one-out analysis for the primary clinic systolic blood pressure meta-analysis, and separate presentation of clinic systolic blood pressure results according to continuous effect metric (final values versus change scores). Binary analyses of blood-pressure target attainment and HbA1c target attainment were treated as exploratory because of sparse data, variable target definitions, and limited comparability across studies.

## Result

### Study selection

**Figure 1:**
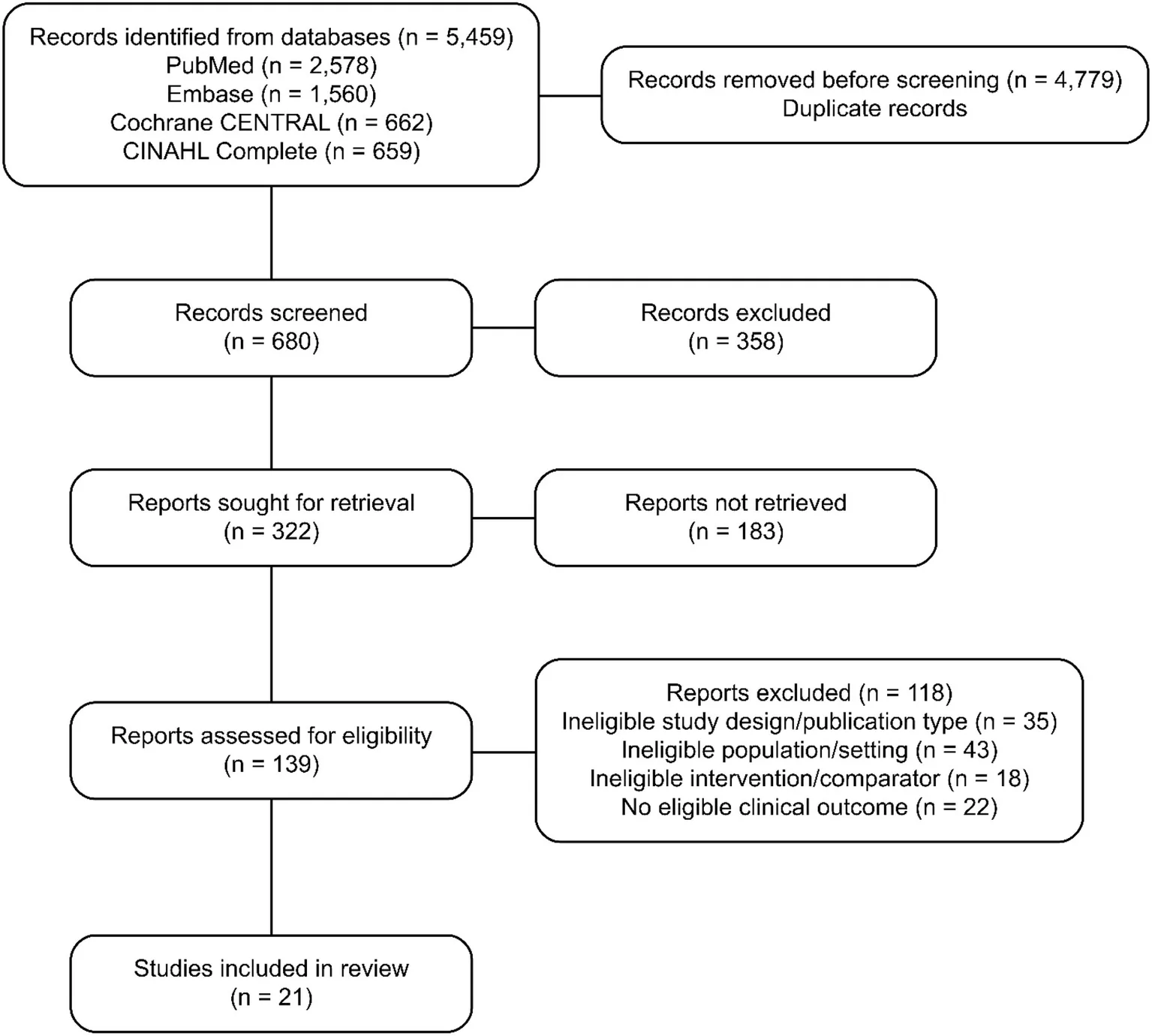
PRISMA 2020 flow diagram of study identification, screening, eligibility assessment, and inclusion. PRISMA 2020 flow diagram of study selection. Of 5,459 records identified from PubMed, Embase, Cochrane CENTRAL, and CINAHL Complete, 21 randomized trials met eligibility criteria. Eligible studies enrolled adult outpatients with hypertension, type 2 diabetes, or mixed cardiovascular-kidney-metabolic (CKM) risk profiles, compared pharmacist-integrated collaborative outpatient care with usual care, and reported extractable outcomes for blood pressure, glycemic control, or related cardiometabolic endpoints. Only English-language published studies were eligible, with no date restriction (PROSPERO: CRD420261363411).

The database search identified 5,459 records, including 2,578 from PubMed, 1,560 from Embase, 662 from Cochrane CENTRAL, and 659 from CINAHL Complete. After removal of 4,779 duplicate records, 680 titles and abstracts were screened, of which 358 were excluded. We sought retrieval of 322 reports, of which 183 were not retrieved, leaving 139 full-text reports assessed for eligibility. Among these, 118 reports were excluded, most commonly because of ineligible study design or publication type (*n* = 35), ineligible population or setting (*n* = 43), ineligible intervention or comparator (*n* = 18), or absence of an eligible clinical outcome (*n* = 22). A total of 21 randomized trials were ultimately included in the review (Figure 1).

### Study characteristics

A total of 21 randomized trials were included, encompassing hypertension, type 2 diabetes, and mixed cardiovascular-kidney-metabolic (CKM) populations. Most were conducted in primary care settings, although several were undertaken in specialty outpatient clinics or community pharmacy-based ambulatory care. The majority evaluated moderate-intensity pharmacist-integrated collaborative care models centered on recommendation-based medication management, whereas a smaller number assessed higher-intensity or protocol-based pharmacist co-management. Follow-up duration varied across studies, with several trials reporting multiple assessment time points. Quantitative usability also varied by outcome, because some studies provided fully analyzable data for continuous or binary pooling, whereas others were limited by incomplete standard deviation reporting, unavailable outcome-level denominators, non-standard comparator structures, or reporting suitable only for narrative or sensitivity analyses (Table 1). Detailed study-level pooling assignments are provided in Supplementary Table S1 and a structured overview of the evidence base by CKM domain and intervention intensity is provided in Supplementary Figure S3.

**Table 1:** Characteristics of included randomized trials and their role in quantitative synthesis.

Study	Country	CKM domain	Design/setting	Pharmacist care model	Follow-up, mo	Analyzed *n* (Int/Ctrl)	Overall RoB 2	Quantitative role
Smith 2016 (19)	United States	HTN	Cluster RCT; primary care	Moderate, recommendation-based collaborative care	9	94/50	Some concerns	Main continuous pool
Aguiar 2018 (20)	Brazil	T2DM	Individual RCT; specialty clinic	Moderate, recommendation-based collaborative care	12	36/37	Some concerns	Partial / limited
Carter 2015 (21)	United States	HTN	Cluster RCT; primary care	Moderate, recommendation-based collaborative care	6, 9, 12, 18, 24	401/224	Some concerns	Main continuous pool
Xiao 2026 (23)	China	T2DM	Cluster RCT; primary care	Moderate, recommendation-based collaborative care	3, 6, 9, 12	291/283	Some concerns	Limited / narrative
Hirsch 2014 (24)	United States	HTN	Individual RCT; primary care	High-intensity, protocol-based co-management	6, 9	75/91	High	Sensitivity only
Chen 2013 (25)	United States	HTN	Cluster RCT; primary care	Moderate collaborative care; ambulatory BP context	6	176/198	High	Secondary quantitative
Alfaraheed 2025 (26)	Jordan	HTN	Individual RCT; primary care	Moderate, recommendation-based collaborative care	3	68/74	Some concerns	Sensitivity only
Halalau 2022 (27)	United States	T2DM	Individual RCT; primary care	Moderate, recommendation-based collaborative care	6, 12	44/42	Some concerns	Main quantitative pool
Javaid 2019 (28)	Pakistan	T2DM	Individual RCT; primary care	Moderate, recommendation-based collaborative care	3, 6, 9	83/52	High	Main glycemic pool; high-risk sensitivity
Lum 2024 (29)	Singapore	T2DM	Individual RCT; mixed outpatient	Moderate, recommendation-based collaborative care	6	70/105	High	Narrative only
Sibounheuang 2025 (30)	Lao PDR	T2DM	Individual RCT; specialty clinic	Moderate, recommendation-based collaborative care	1, 3, 6	64/57	Some concerns	Main quantitative pool
Magid 2013 (31)	United States	HTN	Individual RCT; primary care	High-intensity, protocol-based co-management	6	175/173	Low	Partial / limited
Carter 2008 (32)	United States	HTN	Cluster RCT; primary care	Moderate, recommendation-based collaborative care	2, 4, 6, 8, 9	101/78	Some concerns	Main continuous pool
Carter 2009 (33)	United States	HTN	Cluster RCT; primary care	Moderate, recommendation-based collaborative care	3, 6	192/210	Some concerns	Partial / limited
Jarab 2012 (34)	Jordan	T2DM	Individual RCT; specialty clinic	Moderate, recommendation-based collaborative care	6	77/79	Some concerns	Partial / limited
Hammad 2011 (35)	Jordan	Mixed	Individual RCT; primary care	Moderate collaborative metabolic care	6	110/89	Some concerns	Selected secondary pooling
Green 2008 (36)	United States	HTN	Individual 3-arm RCT; primary care	High-intensity, protocol-based co-management	12	261/258	Low	Limited
Zillich 2005 (37)	United States	HTN	Cluster randomized trial; community pharmacy/outpatient	Pharmacist-intensity comparison; protocol-based involvement	3	64/61	Some concerns	Sensitivity only
Simpson 2011 (38)	Canada	T2DM	Individual RCT; primary care	Moderate, recommendation-based collaborative care	12	131/129	Some concerns	Secondary binary pool
Tobari 2010 (39)	Japan	HTN	Individual RCT; primary care	Moderate collaborative lifestyle-focused care	6	66/66	Some concerns	Partial / limited
Borenstein 2003 (40)	United States	HTN	Individual randomized comparative trial; primary care	Moderate, recommendation-based collaborative care	3, 6, 9, 12	98/99	High	Sensitivity only

CKM, cardiovascular-kidney-metabolic; Ctrl, control; HTN, hypertension; Int, intervention; mo, months; NR, not reported; RCT, randomized controlled trial; RoB 2, Risk of Bias 2; T2DM, type 2 diabetes mellitus.

Intervention-component summaries were conservatively derived from explicitly extracted trial features, including collaborative intensity, medication authority, and directly reported pharmacist-care elements. Quantitative synthesis status reflects validated feasibility for pooling based on the extracted dataset.

### Risk of bias

Overall RoB 2 judgments were low in 2 trials, some concerns in 14 trials, and high in 5 trials. The most common concerns arose from the randomization process, missing outcome data, and selection of the reported result, whereas only a small minority of studies were judged low risk across all domains. Detailed domain-level assessments are provided in Supplementary Table S3, and the full RoB 2 traffic-light plot is shown in Supplementary Figure S5.

The principal pooled quantitative outcomes across the cardiovascular-kidney-metabolic spectrum are summarized in Table 2.

**Table 2:** Summary of quantitative outcomes across the cardiovascular-kidney-metabolic spectrum.

Outcome	Studies (k)	Effect measure	Pooled effect (95% CI)	Heterogeneity	Role in interpretation
Clinic systolic blood pressure, final values	3	MD	−7.11 mm Hg (−9.65 to −4.58)	I^2^ = 0.0%	Primary and most robust finding
HbA1c, continuous	3	MD	−1.26% (−2.36 to −0.16)	I^2^ = 89.2%	Key secondary finding; supportive but less robust
Blood-pressure target attainment	5	RR	1.44 (0.68 to 3.04)	I^2^ = 88.3%	Exploratory; directionally favorable but imprecise
HbA1c target attainment	2	RR	1.62 (0.08 to 31.35)	I^2^ = 0.0%	Supplementary only; too sparse and imprecise for main efficacy interpretation

CI, confidence interval; HbA1c, glycated hemoglobin; MD, mean difference; RR, risk ratio.

### Primary quantitative outcomes

#### Primary hemodynamic outcome: clinic systolic blood pressure

**Figure 2:**
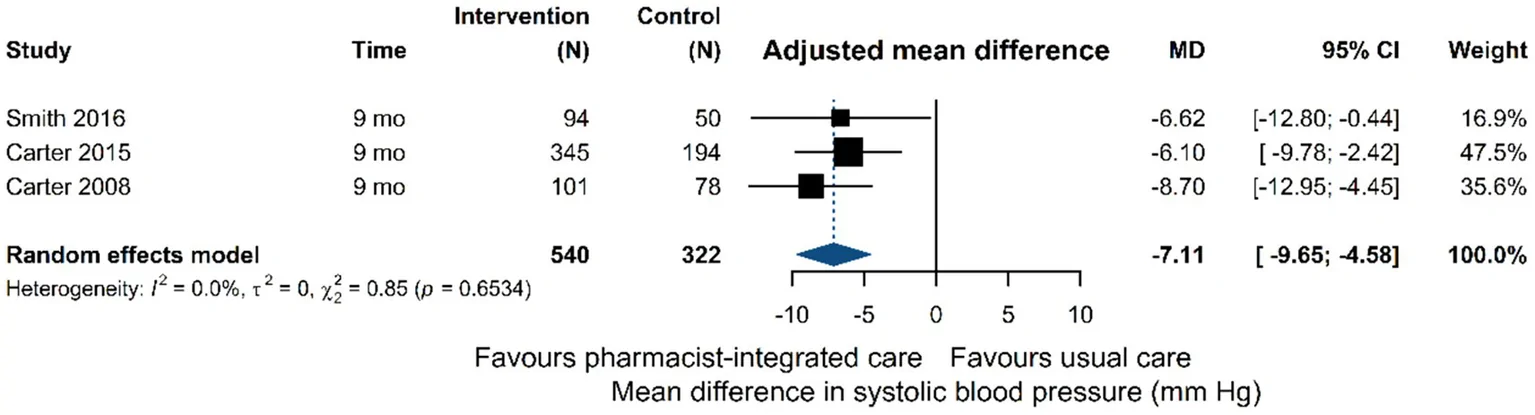
Effect of pharmacist-integrated collaborative care on Clinic systolic blood pressure. Forest plot of clinic systolic blood pressure: pharmacist-integrated collaborative outpatient care versus usual care. Mean differences (MD) and 95% confidence intervals (CI) were pooled using a random-effects model with REML estimation and Hartung–Knapp adjustment. Negative values favor pharmacist-integrated care; positive values favor usual care. Heterogeneity (I^2^) was interpreted using Cochrane Handbook thresholds: 0–40% may not be important, 30–60% moderate, 50–90% substantial, 75–100% considerable. Pooled estimate: MD − 7.11 mm Hg (95% CI − 9.65 to −4.58); I^2^ = 0.0%. Random-effects REML model with Hartung-Knapp adjustment; generic inverse-variance pooling of reported adjusted mean differences.

In the primary continuous hemodynamic synthesis, pharmacist-integrated collaborative care was associated with a significant reduction in clinic systolic blood pressure compared with usual care. Pooling adjusted study-level estimates from Smith 2016, Carter 2015, and Carter 2008 showed a mean difference of −7.11 mm Hg (95% CI −9.65 to −4.58), with no observed between-study heterogeneity (I^2^ = 0.0%). This was the most robust and internally consistent quantitative finding of the review and therefore represents the principal outcome of the CKM-focused evidence synthesis (Figure 2).

#### Key metabolic outcome: HbA1c

**Figure 3:**
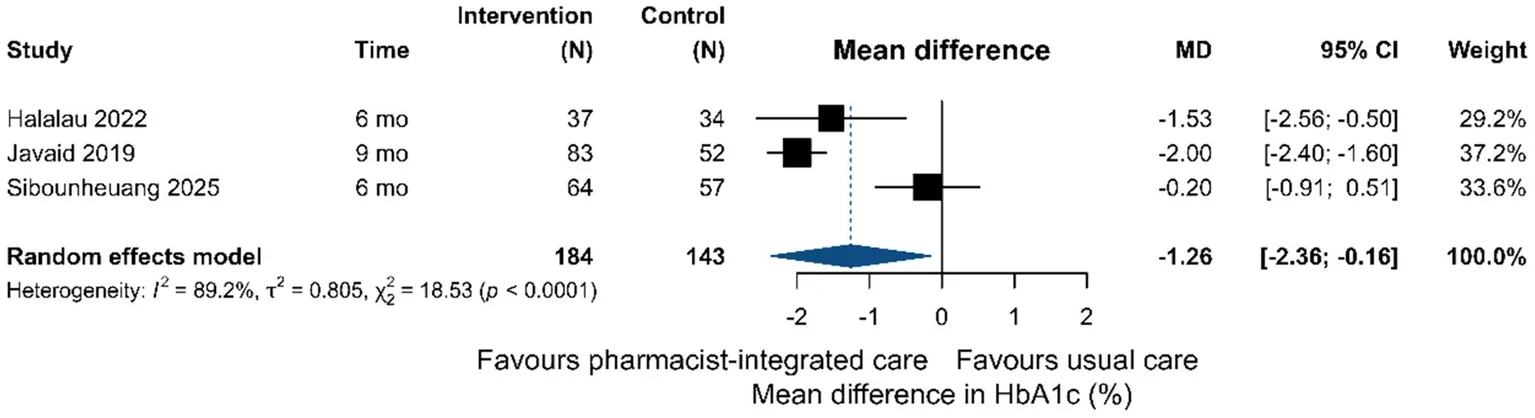
Forest plot of mean differences in HbA1c comparing pharmacist-integrated collaborative care versus usual care. Forest plot of HbA1c: pharmacist-integrated collaborative outpatient care versus usual care. Mean differences (MD) and 95% confidence intervals (CI) were pooled using a random-effects model with REML estimation and Hartung–Knapp adjustment. Negative values favor pharmacist-integrated care. Heterogeneity (I^2^) was interpreted using the Cochrane Handbook thresholds defined in Figure 2. Pooled estimate: MD −1.26% (95% CI −2.36 to −0.16); I^2^ = 89.2%. A prespecified sensitivity analysis excluding the high-risk Javaid 2019 trial attenuated the pooled estimate to MD −0.81% (95% CI −2.11 to 0.49). Primary pool includes analyzable HbA1c rows with complete arm-level mean, SD, and N. Random-effects REML model with Hartung-Knapp adjustment.

For glycemic control, the pooled continuous HbA1c analysis also favored pharmacist-integrated care, with a mean difference of −1.26% (95% CI -2.36 to −0.16). However, heterogeneity was substantial (I^2^ = 89.2%), indicating greater between-study variability than was observed for the primary systolic blood pressure analysis. Moreover, this estimate was sensitive to exclusion of the high-risk Javaid 2019 trial; after removal of high-risk studies, the pooled effect attenuated to −0.81% (95% CI −2.11 to 0.49). Accordingly, the HbA1c finding should be interpreted as supportive but less robust than the primary blood-pressure result (Figure 3).

#### Secondary and exploratory outcomes

Exploratory binary analyses of blood-pressure target attainment showed a directionally favorable effect for pharmacist-integrated collaborative care, but the estimate was imprecise and highly heterogeneous across studies. The pooled risk ratio was 1.44 (95% CI 0.68 to 3.04; I^2^ = 88.3%). Because blood-pressure thresholds differed across trials, the analysis included studies with exploratory or sensitivity-only roles, and one study used a non-usual-care comparator, these findings were considered supportive rather than definitive and are presented in the Supplementary Figure S1.

Evidence for HbA1c target attainment was even more limited. Only two non-high-risk studies contributed to this analysis, yielding a highly imprecise pooled estimate (RR 1.62, 95% CI 0.08 to 31.35). Accordingly, binary glycemic target attainment was treated as a supplementary outcome and was not emphasized in the main efficacy interpretation.

### Sensitivity analyses

Sensitivity analyses supported the robustness of the primary clinic systolic blood pressure finding, which was unchanged after exclusion of high-risk studies because no high-risk trial contributed to that pool. Leave-one-out analysis further showed that omission of any single study did not materially alter the direction of effect for the primary clinic systolic blood pressure outcome, supporting the stability of the main hemodynamic finding (Supplementary Figure S2). To preserve comparability of continuous outcome structures, clinic systolic blood pressure analyses were also examined separately according to effect metric, with final-value and change-score analyses presented independently in the Supplementary Figure S4. In contrast, the continuous HbA1c result was less stable and was attenuated after exclusion of the high-risk Javaid 2019 trial, indicating lower certainty for the glycemic synthesis than for the primary hemodynamic synthesis. Full sensitivity results are provided in Supplementary Table S2.

## Discussion

In this systematic review and meta-analysis of randomized outpatient trials, pharmacist-integrated collaborative care was associated with a clinically meaningful reduction in clinic systolic blood pressure and a more uncertain, though directionally favorable, improvement in HbA1c across CKM-relevant populations. The most robust quantitative finding was the reduction in clinic systolic blood pressure, whereas the glycemic signal was less stable because of substantial between-study heterogeneity and sensitivity to exclusion of a high-risk trial. Exploratory analyses of blood-pressure target attainment were directionally favorable but imprecise, and binary HbA1c target-attainment data were too sparse for firm inference. Taken together, these findings suggest that pharmacist-integrated collaborative outpatient care has its strongest and most consistent evidence base for hemodynamic risk-factor control, with more limited but potentially important benefits for metabolic control.

These findings are highly relevant to the emerging CKM framework. CKM syndrome emphasizes that obesity, diabetes, chronic kidney disease, and cardiovascular disease evolve along a connected continuum and require earlier, more integrated outpatient prevention strategies rather than siloed single-disease management (1, 2). Within this context, blood pressure control remains a central cross-cutting therapeutic target, while dysglycemia and diabetes management contribute to long-term cardiovascular and renal risk modification (3–5). The present findings therefore support pharmacist-integrated collaborative care as a practical model for CKM-oriented outpatient management, particularly where therapeutic inertia, delayed treatment intensification, and medication nonadherence remain major barriers to achieving guideline-recommended targets (6–9).

The strength of the blood-pressure signal in this review is consistent with prior literature showing that pharmacist-involved and team-based care models can improve hypertension control in ambulatory practice (10, 11, 16–18). Earlier systematic reviews demonstrated favorable effects of pharmacist care on cardiovascular risk factors and blood pressure outcomes, while broader community-guide evidence also supports team-based care as an effective strategy for improving blood-pressure control (10, 11, 18). The present review extends that literature by situating these interventions within the CKM framework and by restricting quantitative synthesis to randomized outpatient evidence. Importantly, the primary systolic blood pressure finding was driven by a small but methodologically coherent set of studies using adjusted estimates from collaborative hypertension trials, which likely contributed to the stability of the pooled result (19–21).

From a clinical perspective, the magnitude of systolic blood pressure reduction observed in this review is unlikely to be trivial. Large-scale meta-analytic evidence has shown that even modest reductions in systolic blood pressure are associated with lower risks of major cardiovascular events and mortality, suggesting that a pooled reduction of approximately 7 mm Hg could translate into meaningful downstream benefit if sustained in routine practice (41). This is particularly important for CKM populations, in whom vascular, cardiac, renal, and metabolic risks frequently cluster and amplify one another (1, 2). In that sense, the main blood pressure finding of this review is not only statistically robust but also clinically well aligned with the preventive goals of the target journal collection.

By contrast, the HbA1c findings should be interpreted more cautiously. Although the pooled continuous analysis favored pharmacist-integrated care, heterogeneity was substantial and the estimate attenuated after exclusion of the high-risk Javaid 2019 trial. This pattern suggests that the glycemic benefit may be more context-dependent than the hemodynamic benefit. Several explanations are plausible. Diabetes-focused interventions in the included trials were more heterogeneous with respect to baseline glycemic control, follow-up duration, intervention intensity, outcome reporting, and analytic completeness than the hypertension studies. In addition, some diabetes trials contributed only partially usable or narrative data, which reduced the size and coherence of the quantitative pool. Nonetheless, the overall direction of effect remains consistent with prior pharmacist-led diabetes studies and with the broader concept that collaborative medication management can support safer prescribing, intensification, and monitoring in high-risk patients with type 2 diabetes (12, 14, 15, 23–25). Consistent with this, education-led interventions in patients with type 2 diabetes have similarly shown improvements in medication adherence and quality of life (22), supporting structured patient education as a complementary mechanism within collaborative outpatient care models.

The discussion is also strengthened by considering mechanism. Pharmacist-integrated collaborative care is unlikely to work through a single pathway. Rather, its benefit probably reflects a combination of more frequent therapeutic review, reduced therapeutic inertia, closer adherence support, more rapid adjustment of therapy, reinforcement of self-monitoring, and better continuity between visits (6–9, 12–15). This may help explain why blood pressure responded more consistently than HbA1c: antihypertensive treatment intensification and home blood-pressure monitoring can often be implemented and evaluated over shorter time horizons, whereas glycemic outcomes may be more influenced by baseline disease severity, regimen complexity, and the time needed for stable metabolic response (4, 5, 13–15). For CKM-oriented care pathways, this suggests that pharmacist integration may be especially valuable where rapid outpatient optimization of risk-factor therapy is required.

This review has several strengths. It focused exclusively on randomized outpatient evidence, applied a consistent CKM-oriented conceptual framework, prioritized clinically relevant and methodologically coherent pooled outcomes, and avoided combining noncomparable continuous effect structures in the same model. The primary systolic blood pressure analysis used adjusted study-level estimates and was supported by prespecified sensitivity analyses showing no change after exclusion of high-risk trials. At the same time, several limitations should be acknowledged. First, only a small number of studies contributed to the main quantitative pools. Second, overall risk of bias was not uniformly low, with most studies judged as having some concerns and five judged as high risk. Third, glycemic analyses were limited by heterogeneity, sparse binary outcome data, and incomplete reporting in several otherwise eligible trials. Fourth, although the review was framed within CKM syndrome, the included evidence base was dominated by hypertension and type 2 diabetes trials, with relatively limited representation of kidney-specific populations, renal endpoints, lipid outcomes, or hard cardiovascular events. Finally, the included interventions varied in collaborative intensity, medication authority, monitoring strategy, and comparator structure, which may limit direct generalizability across all outpatient systems.

These limitations point directly to priorities for future research. Next-generation trials should evaluate pharmacist-integrated collaborative care in more explicitly CKM-defined populations, including patients with coexisting diabetes, hypertension, obesity, chronic kidney disease, and established cardiovascular disease. Future studies should also standardize reporting of continuous and target-attainment outcomes, clearly describe collaborative intensity and prescribing authority, incorporate longer follow-up, and capture kidney outcomes, lipid outcomes, hospitalization, cardiovascular events, implementation outcomes, and cost-effectiveness. In addition, modern CKM care increasingly involves more complex drug regimens and monitoring strategies; accordingly, pharmacist-integrated models may become even more relevant as outpatient care moves toward more intensive, multidomain risk-factor management.

## Conclusion

Pharmacist-integrated collaborative outpatient care was associated with a clinically meaningful reduction in clinic systolic blood pressure and a possible improvement in HbA1c across CKM-relevant populations. The blood-pressure finding was the most consistent and methodologically robust result of this review, whereas the glycemic evidence was more heterogeneous and sensitive to study quality. Overall, these findings support pharmacist-integrated collaborative care as a promising outpatient strategy for CKM risk reduction, particularly for hemodynamic control, while underscoring the need for larger, more standardized randomized trials that better reflect the full cardiovascular-kidney-metabolic spectrum.

## Data Availability

The original contributions presented in the study are included in the article/Supplementary material, further inquiries can be directed to the corresponding authors.
