# Supplementary material for: Pharmacist-integrated collaborative outpatient care for risk factor control across the cardiovascular-kidney-metabolic spectrum: a systematic review and meta-analysis of randomized trials
Source: Front Med (Lausanne). 2026 Aug 20;13:1850534. doi: 10.3389/fmed.2026.1850534 (PMC13538878; doi:10.3389/fmed.2026.1850534)
Supplement: Supplementary file 1 [file Table_1.DOCX]

**Supplementary Results**

**Pharmacist-Integrated Collaborative Outpatient Care Across the Cardiovascular-Kidney-Metabolic Spectrum: a systematic review and meta-analysis**

**Supplementary Figure S1. Forest plot of blood-pressure target attainment comparing pharmacist-integrated collaborative care versus control care**

*Forest plot of blood pressure target attainment: pharmacist-integrated collaborative outpatient care versus usual care. Risk ratios (RR) and 95% confidence intervals (CI) were pooled using a random-effects model with REML estimation and Hartung–Knapp adjustment, restricted to studies reporting explicit raw event counts. Target definitions varied across studies (<140/90, <130/80, SBP <130, or "BP control"). RR values greater than 1 favor pharmacist-integrated care; values less than 1 favor usual care. Heterogeneity (I²) was interpreted using the Cochrane Handbook thresholds defined in Figure 2. Pooled estimate: RR 1.44 (95% CI 0.68 to 3.04); I² = 88.3%, indicating substantial-to-considerable heterogeneity. This analysis is exploratory and was not considered a definitive outcome because of the variable target definitions and high between-study heterogeneity.*

**Supplementary Figure S2. Leave-one-out analysis for the primary clinic systolic blood pressure meta-analysis**

Leave-one-out analysis for the validated primary clinic systolic blood pressure pool, restricted to adjusted study-level effect estimates from Smith_2016, Carter_2015, and Carter_2008. Each row shows the pooled adjusted mean difference after omitting one study in turn, alongside the overall pooled estimate. Generic inverse-variance random-effects models with REML estimation and Hartung–Knapp adjustment were used throughout. Negative values favor pharmacist-integrated collaborative care.

**Supplementary Figure S3. Structured subgroup summary of the evidence base by CKM domain and intervention intensity**

Panel A summarizes the studies included by CKM domain; Panel B summarizes the same evidence base by intervention intensity. For each subgroup, the figure displays the number of studies, the principal outcomes contributed, the overall direction of effect, and the study counts classified as main pool, sensitivity/secondary, or narrative only according to the validated study-level pooling assignment. Direction of effect was derived from analyzable outcome rows only and summarized at the study level within each subgroup. This figure is intended as a structured evidence summary and should not be interpreted as a formal subgroup meta-analysis.

**Supplementary Figure S4. Separate pooling of clinic systolic blood pressure by effect metric: final values versus change scores**

Two-panel Forest plot showing separate random-effects pooling of clinic systolic blood pressure according to effect metric. Panel A presents the validated primary final-value analysis using adjusted study-level mean differences from Smith_2016, Carter_2015, and Carter_2008 with generic inverse-variance pooling. Panel B presents the change-score sensitivity analysis using arm-level change data from Hirsch_2014 and Alfaraheed_2025. For harmonization of direction, outcomes reported as reduction from baseline were converted to signed change so that negative values consistently favor pharmacist-integrated collaborative care. REML estimation with Hartung–Knapp adjustment was used throughout.

**Supplementary Figure S5. Risk of Bias 2 traffic-light plot for included randomized trials**

**Supplementary Table S1. Study characteristics and pooling assignment**

| **Study** | **Country** | **CKM domain** | **Design / setting** | **Follow-up, mo** | **Overall RoB 2** | **Main outcomes contributed** | **Pooling assignment** |
| --- | --- | --- | --- | --- | --- | --- | --- |
| Smith 2016 | United States | HTN | Cluster RCT; primary care | 9 | Some concerns | Clinic SBP (final values); DBP supportive | Primary quantitative pool |
| Aguiar 2016 | Brazil | T2DM | Individual RCT; specialty clinic | 12 | Some concerns | HbA1c change reported; SD unavailable | Narrative only |
| Carter 2015 | United States | HTN | Cluster RCT; primary care | 6, 9, 12, 18, 24 | Some concerns | Clinic SBP (final values); DBP supportive | Primary quantitative pool |
| Xiao 2026 | China | T2DM | Cluster RCT; primary care | 3, 6, 9, 12 | Some concerns | HbA1c final values reported; outcome-level N unavailable | Narrative only |
| Hirsch 2014 | United States | HTN | Individual RCT; primary care | 6, 9 | High | SBP change score; DBP supportive | Sensitivity only (high risk) |
| Chen 2013 | United States | HTN | Cluster RCT; primary care | 6 | High | 24-h ambulatory SBP; daytime/night-time ambulatory SBP supportive | Secondary or sensitivity quantitative |
| Alfaraheed 2025 | Jordan | HTN | Individual RCT; primary care | 3 | Some concerns | SBP change score; DBP supportive | Sensitivity only |
| Halalau 2022 | United States | T2DM | Individual RCT; primary care | 6, 12 | Some concerns | HbA1c continuous; HbA1c target attainment; exploratory BP control | Main quantitative pool |
| Javaid 2019 | Pakistan | T2DM | Individual RCT; primary care | 3, 6, 9 | High | HbA1c continuous; exploratory BP target attainment; HbA1c target attainment sensitivity | Main continuous pool; binary sensitivity only |
| Lum 2024 | Singapore | T2DM | Individual RCT; mixed outpatient secondary analysis | 6 | High | Economic/secondary reporting only | Narrative only |
| Sibounheuang 2025 | Lao PDR | T2DM | Individual RCT; specialty clinic | 1, 3, 6 | Some concerns | HbA1c continuous; HbA1c target attainment; exploratory BP control | Main quantitative pool |
| Magid 2013 | United States | HTN | Individual RCT; primary care | 6 | Low | BP continuous outcomes reported without fully usable SD structure | Narrative only or limited appendix |
| Carter 2008 | United States | HTN | Cluster RCT; primary care | 2, 4, 6, 8, 9 | Some concerns | Clinic SBP (final values); 24-h ambulatory SBP secondary; DBP supportive | Primary quantitative pool |
| Carter 2009 | United States | HTN | Cluster RCT; primary care | 3, 6 | Some concerns | BP continuous outcomes partly usable but incomplete for current primary pools | Narrative only or limited appendix |
| Jarab 2012 | Jordan | T2DM | Individual RCT; specialty clinic | 6 | Some concerns | HbA1c change reported without SD; binary counts incomplete | Narrative only |
| Hammad 2011 | Jordan | Mixed CKM | Individual RCT; primary care | 6 | Some concerns | Metabolic syndrome resolution; additional BP and lipid change scores | Supportive single-study quantitative summary |
| Green 2008 | United States | HTN | Individual 3-arm RCT; primary care | 12 | Low | BP outcomes in non-standard multiarm design | Narrative only or limited appendix |
| Zillich 2005 | United States | HTN | Cluster randomized trial; community pharmacy/outpatient | 3 | Some concerns | BP control counts available but comparator was lower-intensity pharmacist care | Sensitivity only (non-usual-care comparator) |
| Simpson 2011 | Canada | T2DM | Individual RCT; primary care | 12 | Some concerns | Exploratory BP target attainment | Exploratory secondary quantitative pool |
| Tobari 2010 | Japan | HTN | Individual RCT; primary care | 6 | Some concerns | Medication reduction outcome; BP counts not explicit | Narrative only |
| Borenstein 2003 | United States | HTN | Individual randomized comparative trial; primary care | 3, 6, 9, 12 | High | Severe post-randomization exclusions | Sensitivity only or narrative |

**Abbreviations:** CKM, cardiovascular-kidney-metabolic; DBP, diastolic blood pressure; HbA1c, glycated hemoglobin; HTN, hypertension; mo, months; RCT, randomized controlled trial; RoB 2, Cochrane Risk of Bias 2; SBP, systolic blood pressure.

**Supplementary Table S2. High-risk-excluded sensitivity analyses**

| **Outcome** | **Main model studies** | **Main pooled estimate** | **High-risk-excluded studies** | **High-risk-excluded pooled estimate** | **Interpretation** |
| --- | --- | --- | --- | --- | --- |
| Clinic systolic blood pressure, final values (MD) | Smith 2016; Carter 2015; Carter 2008 (k=3) | MD -7.11 mm Hg (-9.65 to -4.58); I² = 0.0% | Same as main model (k=3) | Unchanged: MD -7.11 mm Hg (-9.65 to -4.58); I² = 0.0% | No high-risk trial contributed to the primary clinic SBP pool. |
| HbA1c continuous main analysis (MD) | Halalau 2022; Javaid 2019; Sibounheuang 2025 (k=3) | MD -1.26% (-2.36 to -0.16); I² = 89.2% | Halalau 2022; Sibounheuang 2025 (k=2) | MD -0.81% (-2.11 to 0.49); I² = 76.9% | Excluding the high-risk Javaid 2019 trial attenuated the pooled effect and widened the confidence interval. |
| HbA1c target attainment (RR) | Halalau 2022; Sibounheuang 2025 (k=2) | RR 1.62 (0.08 to 31.35); I² = 0.0% | Same as main model (k=2) | Unchanged: RR 1.62 (0.08 to 31.35); I² = 0.0% | The main binary glycemic model was already restricted to non-high-risk trials; Javaid 2019 was reserved for sensitivity only. |

**Abbreviations:** HbA1c, glycated hemoglobin; MD, mean difference; RR, risk ratio; SBP, systolic blood pressure.
**Note:** High-risk trials were defined as studies with overall RoB 2 judged as high. Main clinic SBP and HbA1c target-attainment estimates were aligned to the validated manuscript figures. The high-risk-excluded HbA1c continuous sensitivity estimate was recalculated after removing Javaid 2019 from the main continuous glycemic pool. Effect measures are mean difference (MD) for continuous outcomes and risk ratio (RR) for binary outcomes.

**Supplementary Table S3. Risk-of-bias summary using Cochrane Risk of Bias 2**

| **Study** | **D1. Randomization process** | **D2. Deviations from intended interventions** | **D3. Missing outcome data** | **D4. Measurement of the outcome** | **D5. Selection of the reported result** | **Overall RoB 2** |
| --- | --- | --- | --- | --- | --- | --- |
| Smith 2016 | Some concerns | Low | Some concerns | Low | Some concerns | Some concerns |
| Aguiar 2016 | Low | Some concerns | Some concerns | Low | Some concerns | Some concerns |
| Carter 2015 | Some concerns | Low | Some concerns | Low | Low | Some concerns |
| Xiao 2026 | Some concerns | Low | Some concerns | Low | Low | Some concerns |
| Hirsch 2014 | High | Some concerns | Some concerns | Some concerns | Some concerns | High |
| Chen 2013 | Some concerns | Low | High | Low | Some concerns | High |
| Alfaraheed 2025 | Some concerns | Low | Some concerns | Some concerns | Some concerns | Some concerns |
| Halalau 2022 | Low | Some concerns | Some concerns | Low | Low | Some concerns |
| Javaid 2019 | High | Some concerns | High | Low | Some concerns | High |
| Lum 2024 | Some concerns | Low | High | Low | Some concerns | High |
| Sibounheuang 2025 | Some concerns | Low | Some concerns | Low | Some concerns | Some concerns |
| Magid 2013 | Low | Low | Low | Low | Low | Low |
| Carter 2008 | Some concerns | Low | Low | Low | Some concerns | Some concerns |
| Carter 2009 | Some concerns | Low | Some concerns | Low | Low | Some concerns |
| Jarab 2012 | Some concerns | Low | Some concerns | Low | Some concerns | Some concerns |
| Hammad 2011 | Some concerns | Low | Low | Low | Some concerns | Some concerns |
| Green 2008 | Low | Low | Low | Low | Low | Low |
| Zillich 2005 | Some concerns | Low | Low | Some concerns | Some concerns | Some concerns |
| Simpson 2011 | Low | Low | Some concerns | Low | Low | Some concerns |
| Tobari 2010 | Low | Low | Low | Some concerns | Some concerns | Some concerns |
| Borenstein 2003 | High | Some concerns | High | Low | High | High |

**Abbreviations:** RoB 2, Cochrane Risk of Bias 2; D1, bias arising from the randomization process; D2, bias due to deviations from intended interventions; D3, bias due to missing outcome data; D4, bias in measurement of the outcome; D5, bias in selection of the reported result. **Note:** Overall study judgments across the 21 included trials were **low risk in 2 trials, some concerns in 14 trials, and high risk in 5 trials.** This full domain-level table is best placed in the supplementary appendix, alongside the RoB 2 traffic-light plot, while the main manuscript should retain only the concise numerical summary.
